# Chemical Nature of Electrode and the Switching Response of RF-Sputtered NbO_x_ Films

**DOI:** 10.3390/nano10112164

**Published:** 2020-10-29

**Authors:** Jamal Aziz, Honggyun Kim, Shania Rehman, Muhammad Farooq Khan, Deok-kee Kim

**Affiliations:** Department of Electrical Engineering, Sejong University, 209 Neungdong-ro, Gwangjin-gu, Seoul 05006, Korea; azizjamal37@gmail.com (J.A.); khgking11@gmail.com (H.K.); shania.rehman19@gmail.com (S.R.); mfk@sejong.ac.kr (M.F.K.)

**Keywords:** threshold switching, resistive switching, inert electrode, interfacial oxide layer

## Abstract

In this study, the dominant role of the top electrode is presented for Nb_2_O_5_-based devices to demonstrate either the resistive switching or threshold characteristics. These Nb_2_O_5_-based devices may exhibit different characteristics depending on the selection of electrode. The use of the inert electrode (Au) initiates resistive switching characteristics in the Au/Nb_2_O_5_/Pt device. Alternatively, threshold characteristics are induced by using reactive electrodes (W and Nb). The X-ray photoelectron spectroscopy analysis confirms the presence of oxide layers of WO_y_ and NbO_x_ at interfaces for W and Nb as top electrodes. However, no interface layer between the top electrode and active layer is detected in X-ray photoelectron spectroscopy for Au as the top electrode. Moreover, the dominant phase is Nb_2_O_5_ for Au and NbO_2_ for W and Nb. The threshold characteristics are attributed to the reduction of Nb_2_O_5_ phase to NbO_2_ due to the interfacial oxide layer formation between the reactive top electrode and Nb_2_O_5_. Additionally, reliability tests for both resistive switching and threshold characteristics are also performed to confirm switching stabilities.

## 1. Introduction

Resistive Random Access Memories (ReRAMs) are non-volatile memory devices [1,2] which have attracted considerable attention due to their expediency in scalability [3], high endurance [4], low power consumption [5], and high switching speed [6]. When dealing with such devices in 3D stack arrays, sneak path currents through neighboring cells can easily cause a false readout status for a memory array [1,5]. In order to mitigate this issue, several selector devices were proposed to be integrated with ReRAMs, including tunneling diodes [7], ovonic threshold switching [8], Si-based p-n junction diode [9], mixed ionic electronic conductors [10], and Mott insulators [11].

However, if a single material can fulfill functions of both memory and threshold switching (TS) characteristics, it is of great technological importance in building ReRAMs with a simple cell structure. Niobium oxide (NbO_x_) films are known to exhibit this type of dual electrical response depending on the oxygen stoichiometry, i.e., Nb_2_O_5_ shows the resistive switching [12,13,14], while NbO_2_ shows the threshold switching [15,16]. NbO_2_ and other related oxides such as VO_2_ are Mott insulators, which exhibit insulator to metal transition (IMT) due to current-induced Joule heating [11,15,17,18,19]. However, for several decades, there has been a controversy on whether this phase transformation is caused by a low temperature (<500 K) non-linear transport driven conduction mechanism [20,21] or high temperature Mott metal-insulator transition [11,17,18]. Assuming that temperature manifested by joule heating is increased by just 100–200 K, Slesazeck et al. [22] developed a physical model which describes the threshold switching effect in NbO_2_-based filamentary switching devices by a temperature activated Poole–Frenkel (P–F) conduction. Mott insulators have been extensively studied due to their high switching speed [19] with low transition energy and reliable switching performance [6,18,23]. Although NbO_2_ exhibits excellent TS behavior and is thermodynamically very stable [24] in comparison to VO_2_ [19], but its lower off-state resistance hinders its application in cross-point arrays. This response is due to the segregation of oxygen vacancies across grain boundaries in oxide-based systems and forms the conducting leakage path by forming a nano-filament [25]. To solve this problem, several researchers proposed inserting a metal oxide layer between the electrode and NbO_x_ layer to reduce the leakage current as it suppresses the interface defects, resulting in higher Schottky barrier height [17,26,27].

Understanding the role of different electrodes on the switching properties provides useful measures for the optimization of switching performance of the active layers. Even for the same active layer, the switching behavior was found to vary depending on the electrode type used [28,29,30]. Use of an inert electrode did not alter the stoichiometry of the active layer and has very poor switching uniformity due to the oxygen runout during electroforming and programming processes [28], while for the case of reactive electrode, improved switching can be achieved by an interfacial oxygen barrier layer formed as a result of the interfacial reaction between the top electrode (TE) and the active layer [29,30].

Our present study focused on the investigation of the effects of various TE materials on the electrical properties of RF-sputtered NbO_x_ films. The significance of the reaction at TE/NbO_x_ interface is demonstrated on the basis of Gibb’s free energy of oxide formation of different TE metals. Raman Spectroscopy was performed to analyze the structural phase of as-grown NbO_x_ films, whereas X-ray photoelectron spectroscopy (XPS) depth profiling was performed to provide a specific chemical composition of the NbO_x_ films at different depths influenced by different TEs.

## 2. Experimental Details

Oxidized silicon substrates were chosen for the fabrication of switching devices. Firstly, a 10-nm-thick Ti adhesive layer and then a 60-nm-thick Pt layer were deposited by the evaporation technique to function as a bottom electrode (BE). After the deposition of BE, NbO_x_ films of 60 nm thickness were synthesized using a 2-inch Nb metal target in a RF-sputtering unit by giving 130 W power, and the stoichiometry of NbO_x_ was controlled by the ratio of argon to oxygen (13/5 at 18 sccm) at 500 °C. After the successful deposition of an active layer, the island structure was fabricated using shadow mask of size 75 × 75 µm^2^ during niobium (Nb) and tungsten (W) TE deposition of thickness 60 nm in RF-sputtering unit to make metal-oxide-metal device structures. This deposition was achieved by giving a forward power of 120 W with an Ar Flow of 15 sccm using a 2-inch metal target, whereas gold (Au) TE of thickness 60 nm was deposited in an e-beam sputtering at room temperature. The base pressure for the deposition of an active layer and TE was 2.6 × 10^−4^ Pa and the working pressure was 3.6 Pa. The thickness of each layer was verified by an Alpha Step D-600 stylus profiler. Finally, the electrical transport measurement of the devices was carried out with an Agilent B1500 semiconductor parametric analyzer at room temperature in air. In order to find out the switching speed of threshold switching devices, a pulse generator unit (PGU) was used to supply the voltage pulses of 50 ns duration and an oscilloscope (Keysight Technologies, Santa Rosa, CA, USA) was used to monitor the input pulse and the output response simultaneously during the device SET operation (transition from insulating to highly conducting state).

## 3. Results and Discussion

**Figure 1:**
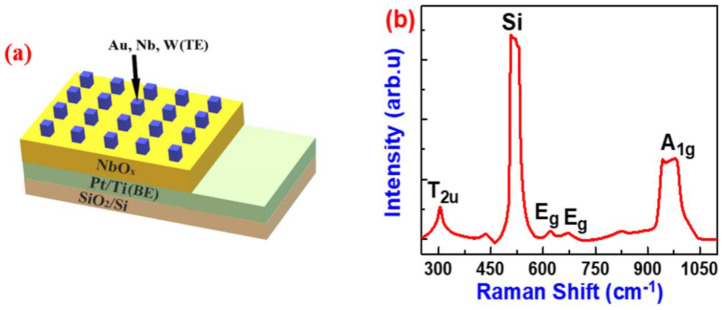
(**a**) Schematic illustration of NbO_x_-based devices. (**b**) Raman shift spectra of as-prepared NbO_x_ with Pt as a bottom electrode (BE) measured at room temperature.

Supplementary Data Figure S1a shows the cross-sectional scanning electron microscopic (SEM) image of the NbO_x_/Pt devices prepared in the RF-sputter. The thickness of SiO_2_, Ti/Pt and NbO_x_ was ~100, 70 and 60 nm, respectively. The surface morphology of the NbO_x_ films was confirmed from Atomic force microscopic (AFM) images shown in Supplementary Data Figure S1b. Root means square roughness (RMS) calculated from the AFM images was ~25 nm indicating a rough surface and grain size (GS) calculated from the images was ~49 nm. Rough surfaces and ~49 nm larger GS are better for switching probability and low leakage currents, respectively. Figure 1a shows a schematic illustration of NbO_x_ devices with 3 different TEs employed in the study. The voltage bias was applied to the TE, while the BE was grounded throughout the measurement. For the determination of the film composition of the NbO_x_ active layer, we conducted Raman spectroscopy of as-prepared NbO_x_ samples without TE, as shown in Figure 1b. A plethora of work has been performed in order to discover the vibrational modes of different oxides of niobium [12,31,32,33,34]. Our Raman results showed all peaks corresponding to Nb_2_O_5_, indicating that it is the dominant phase in the as-prepared oxide films. Spectrum shows 4 clear Raman peaks at 304, 625, 670 and 990 cm^−1^. The Raman peak at around 990 cm^−1^ is a longitudinal optical (LO) A_1g_ mode, which appears due to the symmetric single stretching vibration mode of Nb and O double bond (Nb = O). This mode is the characteristic mode of most oxide systems. In the range of 600–700 cm^−1^, we observed two transverse optical (TO) E_g_ stretching modes of Nb-O single bonds. In the range of 200–300 cm^−1^, we observed T_2µ_ mode (T is triply degenerated modes). These modes appeared due to the angle deformations related to Nb-O-Nb bonds. The broad nature of the bands could suggest that the prepared oxide was amorphous [35]. Our results are in accordance with the previous reports [32,33,34,35].

**Figure 2:**
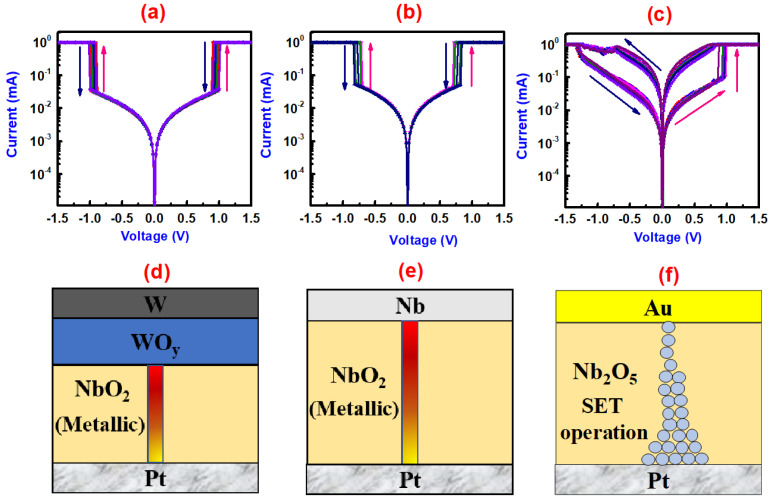
Electrical measurements of different top electrode (TE) devices (**a**) W/NbO_x_/Pt stack, (**b**) Nb/NbO_x_/Pt stack and (**c**) Au/NbO_x_/Pt stack. Schematic diagrams of SET operation of different TE devices (**d**) Poole–Frenkel (P–F)-induced threshold switching (TS) due to the presence of interfacial WO_y_-NbO_2_ bilayer structure formed after electroforming due to the reduction of the Nb_2_O_5_ active layer by W TE, (**e**) P–F induced TS due to the presence of NbO_2_ phase formed by the reduction of the Nb_2_O_5_ active layer by Nb TE, (**f**) Formation of oxygen vacancies filament in oxygen deficient Nb_2_O_5_ films during + ive bias applied to the Au TE.

Figure 2 shows the electrical switching and the schematic diagrams of the operating mechanisms of NbO_x_ films with three different TEs. The as-prepared (W, Nb, Au) /NbO_x_/Pt devices were in high resistance state (HRS). An initial forming process was required to change the phase of the films. After the required electroforming process, threshold switching was observed for the case of W and Nb TEs, while bipolar resistive switching was observed for the case of the Au TE devices.

Supplementary Data Figure S2a–c shows the forming process of W/NbO_x_/Pt, Nb/NbO_x_/Pt and Au/NbO_x_/Pt stack, respectively. For the forming process, a voltage of 6 V with a compliance current of 1 mA was applied. Forming voltage observed for W TE devices (5.5 V) was higher than Nb TE devices (3.5 V). After the required forming process, when the applied voltage was swept from 0 to 1.5 V, the current shows discontinuous behavior with an abrupt increase in current at threshold voltage (V_th_ = 1.0 V for W TE and 0.84 V for Nb TE) as shown in Figure 2a,b. The current reaches the compliance current (1mA in this case), which is applied to avoid any damage to the device. This abrupt increase in current corresponds to temperature activated Poole–Frenkel conduction mechanism [15,17,18,36]. The device maintains this limiting current value till 1.5 V, and when the voltage is decreased, the device still shows a maximum current to its output until the hold voltage (V_h_ = 0.9 V for W TE and 0.72 V for Nb TE). Below this voltage, device represents abrupt decrease in its current value and the device is in the OFF state. Symmetric behavior was also observed in the negative voltage sweeping, consistent with the non-polar nature of thermally induced Joule heating. 

Figure 2c shows the typical current–voltage (I–V) characteristics of Au/NbO_x_/Pt stack under consecutive voltage bias sweeping. Initially, the devices were in the HRS and the forming process, which changes the HRS of the device to reversible switching states, was manifested and takes place at ~5.85 V under a compliance current of 1 mA shown in Figure S2c. After the electroforming process, the device was in the low resistance state (LRS). When the bias polarity is reversed, the current gradually decreases at ~−1.3 V. This increased resistance of the device is referred to as a RESET process. When a device is positive biased after RESET process, the current increases sharply at ~0.96 V, attributable to the decrease in the device resistance. This state of the device is referred as a SET process. These SET and RESET processes were reproducible, and no significant degradation in the memory window and switching voltage of the device was observed.

The forming process was found to be compulsory to exhibit the threshold and the resistive switching of the niobium oxide films in this study. The different forming and threshold switching characteristics of W and Nb TE devices are presumably attributed to the different reactivity of TE. Park et al. [37] reported that the sputter grown films are amorphous in nature, and therefore electroforming was needed to form the crystalline tetragonal phase of NbO_x_ films within the amorphous matrix to exhibit threshold switching. Moreover, in the present case, reduction of the Nb_2_O_5_ phase to NbO_2_ was also necessary to exhibit this abrupt electrical response. The change in the forming voltage is expected to be linked to the oxygen affinity of electrodes and change in the Gibb’s free energy (ΔG) governing the interfacial reaction between TE and oxide layer to reduce the Nb_2_O_5_ films to NbO_2_ for threshold switching [38]. The ΔG of the reactions of W and Nb with niobium oxides are shown in Table S1 [39]. For the case of W TE devices, all the reactions between W and niobium oxides have positive ΔG; hence the energy source for the reactions to occur is given by high voltage, which is correlated with the field induced electromigration of oxygen ions inside the W layer [40]. For the case of Nb TE devices, as the oxygen affinity of Nb metal is much higher than the oxygen affinity of W metal [41], Nb TE can more easily reduce NbO_x_ films than W TE. Furthermore, as the reactions between Nb and niobium oxides have negative ΔG (Table S1), it reduces Nb_2_O_5_ to NbO_2_ during sputtering because of its spontaneity. Hence, the forming process for the Nb TE device was required for the crystallization of NbO_x_ films resulting in a lower forming voltage than W TE devices. Additionally, the WO_y_ interface resistor layer in W TE devices results in a larger threshold voltage than Nb TE-based NbO_x_ devices, which is consistent with the results of Park et al. [27] by introducing NiO_y_ layer. For the case of resistive switching devices, electroforming was required for the oxygen vacancy creation process in Nb_2_O_5_ devices. Under high positive voltage, O^2-^ ions drift towards the anode where they evolve as oxygen gas, thus creating oxygen vacancies in the Nb_2_O_5_ films [40]. After the forming process, resistive switching is caused by the formation (SET) or rupture (RESET) of conducting filament of oxygen vacancies in oxygen-deficient Nb_2_O_5_ films. Our results are consistent with the previously reported Nb_2_O_5_ ReRAM [14]. Schematics were drawn in Figure 2d–f to demonstrate the different switching modes of W, Nb and Au TE-based NbO_x_ devices.

Based on the above results of Nb and W TE devices showing threshold switching (TS), physical modelling of low current behavior consisting of current controlled negative differential resistance (NDR) is required which is shown in Supplementary Data Figure S3. For this purpose, a series resistor (R_ser_) is inserted between a threshold switching device V_NbO2_ and the voltage source (inset of Figure S3a). At minimal voltages, most of the voltage is drop across TS device (V_NbO2_) as its resistance is much higher than R_ser_. When the threshold voltage is reached, a consequent amplification across the series resistor equal to V_s_ + ΔV_NDR_ is developed due to voltage drop equal to V_NbO2_-ΔV_NDR_ across NbO_x_ TS device. Thus, TS and R_ser_ built a voltage divider stabilizing the NDR region of TS device. The starting point of the NDR region and its curvature define the hysteresis in the circuit without R_ser_. The particular phase transition at lower currents has been accepted as result of field induced thermal runaway process induced by self-heating. To model the conduction process below threshold, different mechanism were fitted where Poole–Frenkel conduction provides the best fit to the current controlled sweep (Supplementary data Figure S3b) indicating that IMT is not the dominant mechanism behind the threshold switching effect rather it was Poole–Frenkel-induced threshold switching effect [22].

In order to determine the chemical composition of NbO_x_ films influenced by different TEs, XPS depth profiling spectra of different TEs-based structures was performed after electroforming and consecutive I–V sweeps. The depth profiling spectra of Au and Nb elements in Au/NbO_x_/Pt stack is shown in Figure S4. Nb spectra at different etch times from 1 to 75 min in Figure S4a showed different oxide phases of niobium. According to the fitted results at the interface in Figure S4b, two distinct peaks were observed with a peak position of ~206.50 and ~209.50 eV corresponding to the binding energy (B.E) of NbO_2_(3d_5/2_) and Nb_2_O_5_(3d_3/2_), respectively [30,42]. A red shift in the B.E of Nb 3d (Δ = 0.3 eV) has been observed on etching (10 min to 55 min) in Figure S4a which corresponds to the increase in oxygen vacancies in the films. The Au spectra at the interface in Figure S4c showed two peaks at ~83.5 and ~87.1 eV, resulting from the electron ejection from the core orbit 4f, of 5/2 and 7/2, respectively [43]. Depth profile confirms that no interfacial reaction of Au has occurred and Nb_2_O_5_ is a dominant phase at the interface and the bulk as shown in Figure S4b,d, respectively.

**Figure 3:**
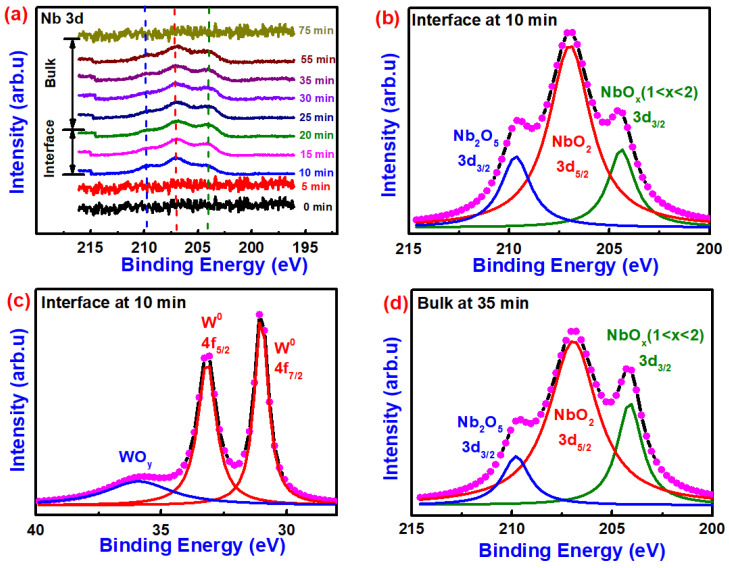
XPS Depth Profiling spectra of Nb and W elements in W/NbO_x_/Pt. (**a**) Nb 3d at different etch times. Fitted results of (**b**) Nb and (**c**) W spectra at 10 min etch time which shows the formation of WO_y_-NbO_x_ interfacial bilayer. (**d**) Fitted results of Nb spectra at 35 min etch time show the coexistence of various oxides of niobium in the bulk.

The depth profiling spectra of W and Nb elements in W/NbO_x_/Pt stack is shown in Figure 3. Nb spectra at different etch times from 1 to 75 min shown in Figure 3a indicates the existence of multiple oxide phases of niobium. According to the fitted results at the interface in Figure 3b, three peaks were observed with peak positions ~204.50, ~206.45 and ~209.70 eV which corresponds to the B.E of NbO_x_ (1 < × < 2) (3d_3/2_), NbO_2_(3d_5/2_) and Nb_2_O_5_(3d_3/2_) peaks, respectively [30,42]. The W 4f spectra represent three clear peaks. The first characteristic doublet with a peak position around 31.5 eV (W 4f _7/2_) and 33.7 eV (W 4f _7/2_) corresponds to W^0^ oxidation state whereas a broad peak around 35.7 eV corresponds to the oxidation of W occurring at the interface. Furthermore, detailed fitting results of Nb 3d and W 4f at the interface, shown in Figure 3b,c, respectively, indicate that at the depth level of 10 min etching, WO_y_-NbO_x_ interfacial bilayer was preliminarily found. As all the reactions between W and niobium oxides have positive ΔG (Table S1), it is hypothesized that the formation of WO_y_ layer occurred as a byproduct during electroforming, which correlates with the field induced electromigration of oxygen ions inside the W layer. Furthermore, after electroforming and consecutive I–V sweeps, oxygen from the Nb_2_O_5_ diffuses into W, oxidizing it further and resulting in the reduction of Nb_2_O_5_ to NbO_2_ and then NbO_x_ (1 < × < 2) This is the reason that the NbO_2_ phase is the dominant phase at the interface and at the bulk which results in the threshold switching of such devices [11]. Reactions possible for the reduction of Nb_2_O_5_ and NbO_2_ by W TE are shown in reaction Equations (1) and (2), respectively.

W + 2 Nb_2_O_5_ → WO_2_ + 4 NbO_2_ (1)

W + 2 NbO_2_ → WO_2_ + 2 NbO (2)

The depth profiling spectra of Nb 3d in Nb/NbO_x_/Pt stack are shown in Figure S5. Figure S5a shows the typical Nb 3d spectra at different etch times from 1 to 75 min. Likewise, W TE-based structures, multiple oxide phases of niobium also coexist at the interface with peak positions of ~203.60, ~206.20 and ~209.10 eV, corresponding to the binding energies of NbO_x_ (1 < × < 2) (3d_3/2_), NbO_2_(3d_5/2_) and Nb_2_O_5_(3d_3/2_) peaks, respectively. No metallic peak of the Nb^0^ ionic state has been observed. As all the reactions between Nb and niobium oxides have negative ΔG (Table S1), the reduction from Nb_2_O_5_ to NbO_2_ by Nb TE is expected to have occurred during the Nb TE deposition process. Additionally, after electroforming and consecutive I–V sweeps, NbO_2_ reduces further to NbO_x_ (1 < × < 2) owing to its high oxygen affinity. Detailed fitting results of Nb 3d at the interface and at the bulk are shown in Figure S5b,c, respectively. As discussed by Lin et al. [42], when the NbO_2_ is under oxidized, its binding energy peak value is blue shifted; considering NbO_2_ peak of Au TE structure as a reference peak, the blue shift of NbO_2_ peak at the interface in W TE devices (Δ = 0.05 eV) is less than the Nb TE devices (Δ = 0.3 eV), which is attributed to its higher oxygen affinity than W. Moreover, for Nb TE devices, NbO_2_ is a dominant phase at the interface and at the bulk which results in the threshold switching of such devices. Reactions possible for the reduction of Nb_2_O_5_ and NbO_2_ by Nb TEs are shown in Equations (3) and (4), respectively.

Nb + 2 Nb_2_O_5_ → 5 NbO_2_ (3)

Nb + NbO_2_ → 2 NbO (4)

Considering the Figure 3 of W/NbO_x_/Pt stack and Supplementary Data Figures S4 and S5 with Au and Nb TE devices, respectively, the XPS depth profiling both at the interface and the bulk confirms the Nb_2_O_5_ prominent phase for Au TE-based devices while NbO_2_ for both Nb TE and W TE-based devices.

Comparative XPS depth analysis between the W, Nb and Au TE-based devices, suggests that the Nb and W reduces the entire film to Nb_2_O_5-x_ and other sub-oxide phases with NbO_2_ as a prominent phase whereas Nb_2_O_5_ as the prominent phase for Au TE devices observed at all etch times. As shown in Table S1, as the reactions between Nb and niobium oxides NbO_x_ have negative ΔG (Table S1), it reduces Nb_2_O_5_ to NbO_2_ during sputtering because of its spontaneity. When the NbO_x_ matrix came into contact with metallic Nb, oxygen from NbO_x_ diffused into Nb due to high energy during the deposition process, while for the case of W TE-based devices, reactions between W and niobium oxides have positive ΔG; hence the energy source for the reactions to proceed is required which is given by high electroforming voltage, which is correlated with the field-induced electromigration of oxygen ions inside the W layer.

**Figure 4:**
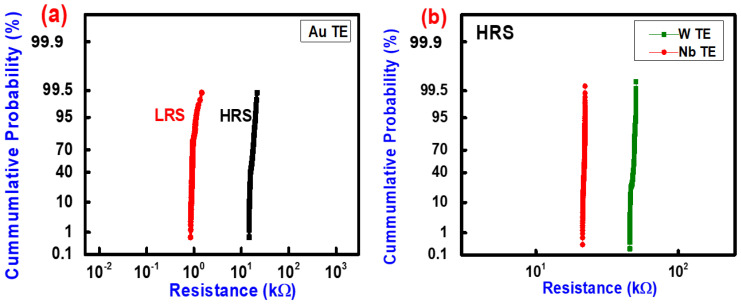
(**a**) The cumulative probability distribution of high resistance state (HRS) and low resistance state (LRS) of Au/NbO_x_/Pt device. (**b**) The cumulative probability distribution of HRS @ 0.20 V of switching Cycles of W/WO_y_/NbO_x_/Pt and Nb/NbO_x_/Pt devices.

In order to stack these selector devices with ReRAM to achieve high density data storage implementation, the state stability of the selector devices needs to be high. For comparison, we measure 10 devices of each electrode (W, Nb, Au), and we have inferred that the W/WO_y_/NbO_x_/Pt devices showed more reproducibility and stability than (Nb, Au)/NbO_x_/Pt devices. The threshold response of the devices with W TE was not degraded instead of high compliance current (1 mA) in the ON state (Figure 4b). In contrast, filamentary ReRAM shows poor switching uniformity because of the randomly formed and ruptured oxygen vacancies filament and oxygen runout through TE [29,40] due to there being no barrier layer, whereas the NbO_2_ threshold device shows a good switching uniformity due to Poole–Frenkel-induced threshold switching, and even more stability due to oxygen barrier layer for the case of the W reactive electrode [28,29,30].

**Figure 5:**
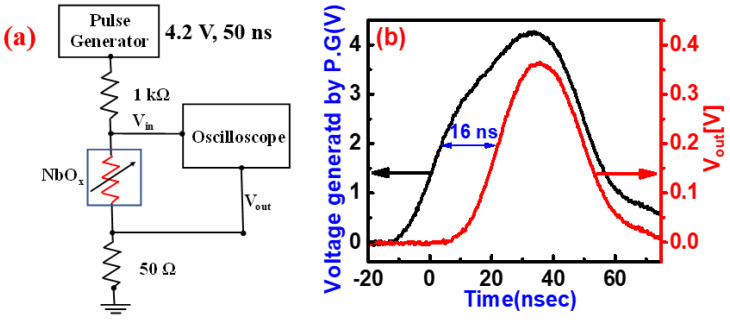
(**a**) Circuit Configuration of W/WO_y_/NbO_x_/Pt device speed measurement, (**b**) Real-time oscilloscope measurements during the SET pulse condition.

As discussed earlier, these W-based NbO_x_ threshold devices showed excellent stability and uniformity, and it is very crucial in practical applications that the selector device should operate under pulse mode with high switching speed ability to transform from insulator (OFF) to metal (ON). In order to find the switching speed of these W/WO_y_/NbO_x_/Pt devices, we monitored input pulse and the output response of the device simultaneously during SET operation using an oscilloscope to make real-time measurements. The circuit configuration for the switching speed measurement is shown in Figure 5a. For this purpose, a series resistor of 1kΩ is inserted between the threshold switching device (NbO_x_) and the pulse generator (P.G) while an oscilloscope is connected across the NbO_x_ TS device to record its real-time transient response. When an input pulse of 4.2 V and 50 ns duration was applied, the device was switched from OFF to ON state in 16 ns as shown in Figure 5b. This fast transition was induced from the Poole–Frenkel conduction by both high electric fields and thermal energy [12,19,31]. Furthermore, an NDR is developed across the NbO_x_ TS due to the series resistance of 1 kΩ. This will result in a consequent amplification across the series resistor equal to (V_S_ + ΔV_NDR_), where V_S_ is the voltage drop across the series resistor. This is the reason how internal amplification in hyper field effect transistors is possible by connecting them with TS devices [44]. This is also discussed in Supplementary data Figure S3. These NbO_x_ devices with W TE and WO_y_ barrier layers show a switching speed that is fast enough for its usage as a selector with any ReRAM device and in many other electrical applications.

## 4. Conclusions

In summary, we found that a simple NbO_x_ stack can provide both switching and memory characteristics. With the Au inert TE, the resistive switching of Nb_2_O_5_ was observed with good memory performance, whereas the threshold switching was observed for W and Nb reactive electrodes due to the reduction of NbO_x_ films from Nb_2_O_5_ to NbO_2_. XPS depth profiling suggests that the entire film was reduced to lower oxide phases of Nb when in contact with W and Nb electrodes. Stable and reproducible threshold switching with minimal memory window degradation was observed in W/NbO_x_/Pt devices, which was attributed to the formation of a WO_y_ interface layer with NbO_2_. The transient measurements of W TE-based devices show a threshold switching speed of 16 ns that is fast enough for its use as a selector with any ReRAM device. Such a simple metal oxide metal (MOM) structure with concurrent memory switching and threshold switching allows for the construction of high density cross point memories by solving the sneak path problems.

## Supplementary Materials

The following are available online at https://www.mdpi.com/2079-4991/10/11/2164/s1, Table S1: Possible chemical reactions between niobium oxides and metals (W or Nb) with change of the Gibbs potentials ΔG of the reaction equations; Figure S1: (a) Cross-sectional SEM image of NbOx/Pt devices, (b) AFM images of as-grown NbOx films with root mean square roughness (RMS) and the grain size (GS) value shown; Figure S2: Electroforming process of simple NbOx/Pt stack with 3 different top electrodes. (a) W, (b) Nb and (c) Au; Figure S3: (a) Measured current-voltage characteristics of W/WOy/NbOx/Pt devices under current controlled operation (inset shows the measurement circuit with series resistor), (b) Poole-Frenkel fit to the current controlled sweep in the linear region below threshold voltage; Figure S4: XPS depth Profiling spectra of Nb and Au elements in Au/NbOx/Pt. (a) Nb 3d at different etch times. Fitted results of (b) Nb and (c) Au spectra at 10 min etch time which shows no oxide interfacial bilayer. (d) Fitted results of Nb spectra at 35 min etch time show the coexistence of various oxides of niobium in the bulk; Figure S4: XPS depth Profiling spectra of Nb and Au elements in Au/NbOx/Pt. (a) Nb 3d at different etch times. Fitted results of (b) Nb and (c) Au spectra at 10 min etch time which shows no oxide interfacial bilayer. (d) Fitted results of Nb spectra at 35 min etch time show the coexistence of various oxides of niobium in the bulk; Figure S5: XPS Depth Profiling spectra of Nb element in Nb/NbOx/Pt. (a) Nb 3d at different etch times. Fitted results of (b) Nb spectra at 10 min etch time and (c) Nb spectra at 35 min etch time show the coexistence of various oxides of Niobium.
