# Supplementary material for: Chemical Nature of Electrode and the Switching Response of RF-Sputtered NbOx Films
Source: Nanomaterials (Basel). 2020 Oct 29;10(11):2164. doi: 10.3390/nano10112164 (PMC7693469; doi:10.3390/nano10112164)
Supplement: Supplementary file 1 [file nanomaterials-10-02164-s001.pptx]

## Slide 1
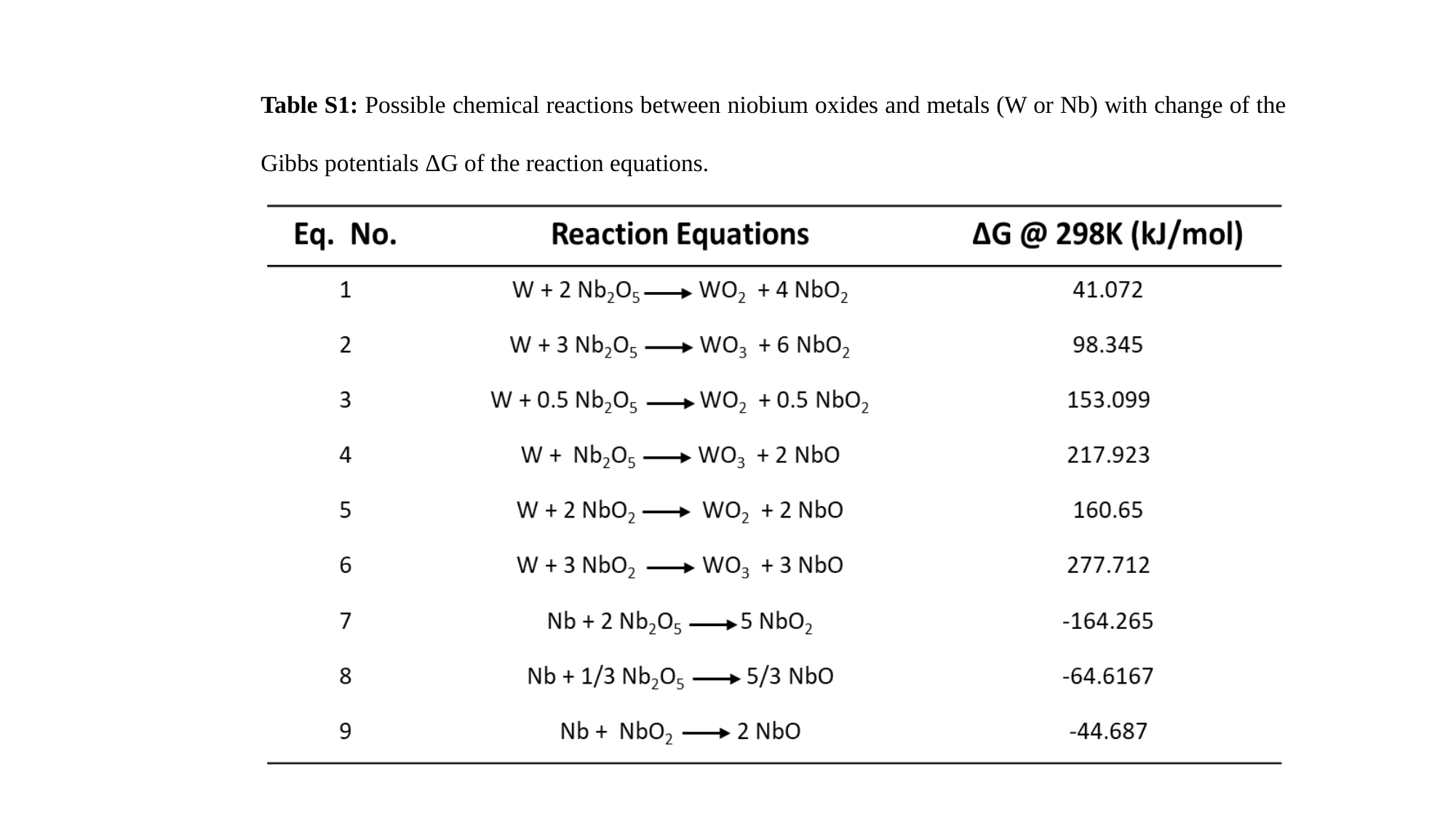

Table S1: Possible chemical reactions between niobium oxides and metals (W or Nb) with change of the Gibbs potentials ΔG of the reaction equations.

## Slide 2
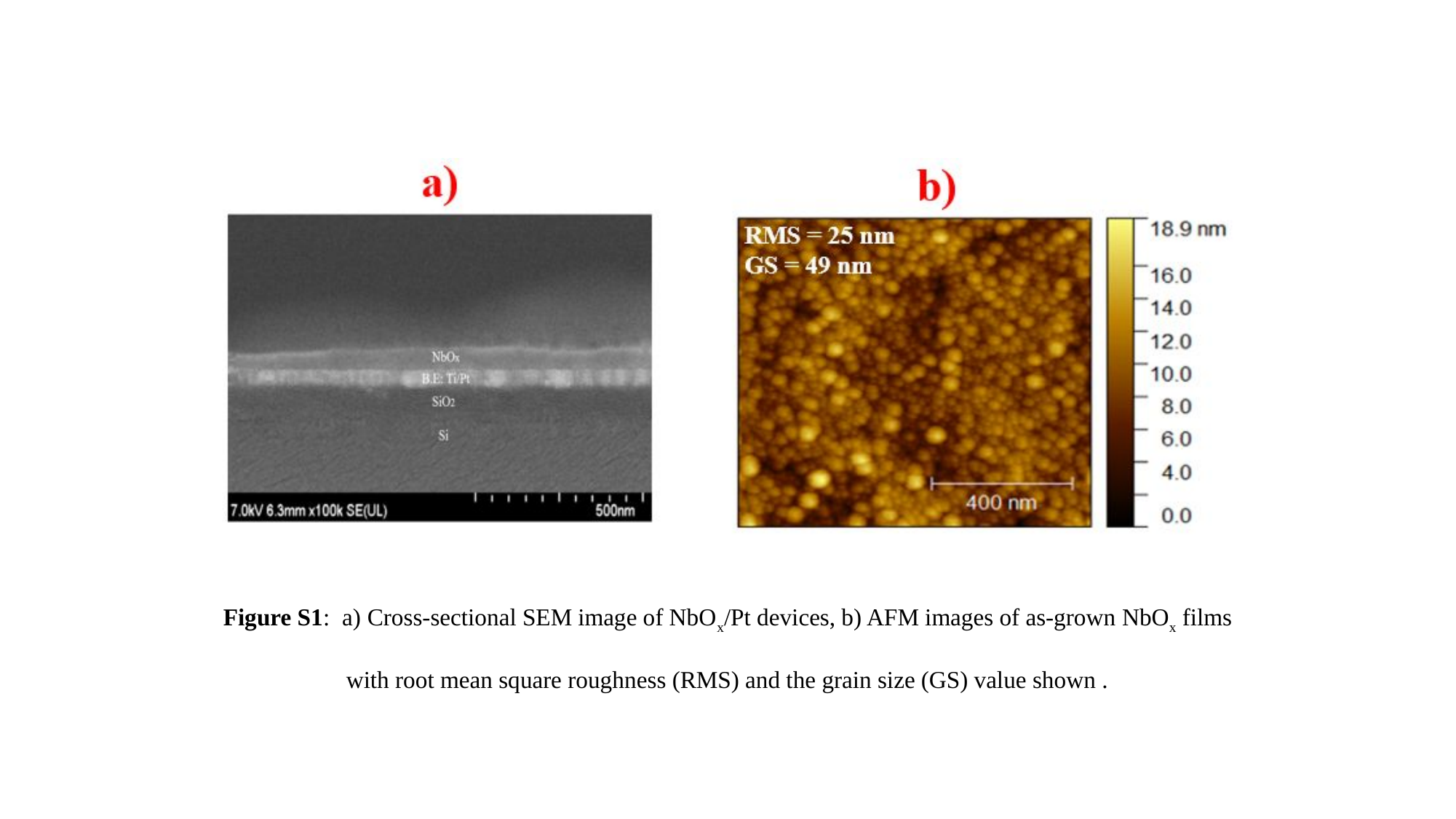

Figure S1: a) Cross-sectional SEM image of NbOx/Pt devices, b) AFM images of as-grown NbOx films with root mean square roughness (RMS) and the grain size (GS) value shown .

## Slide 3
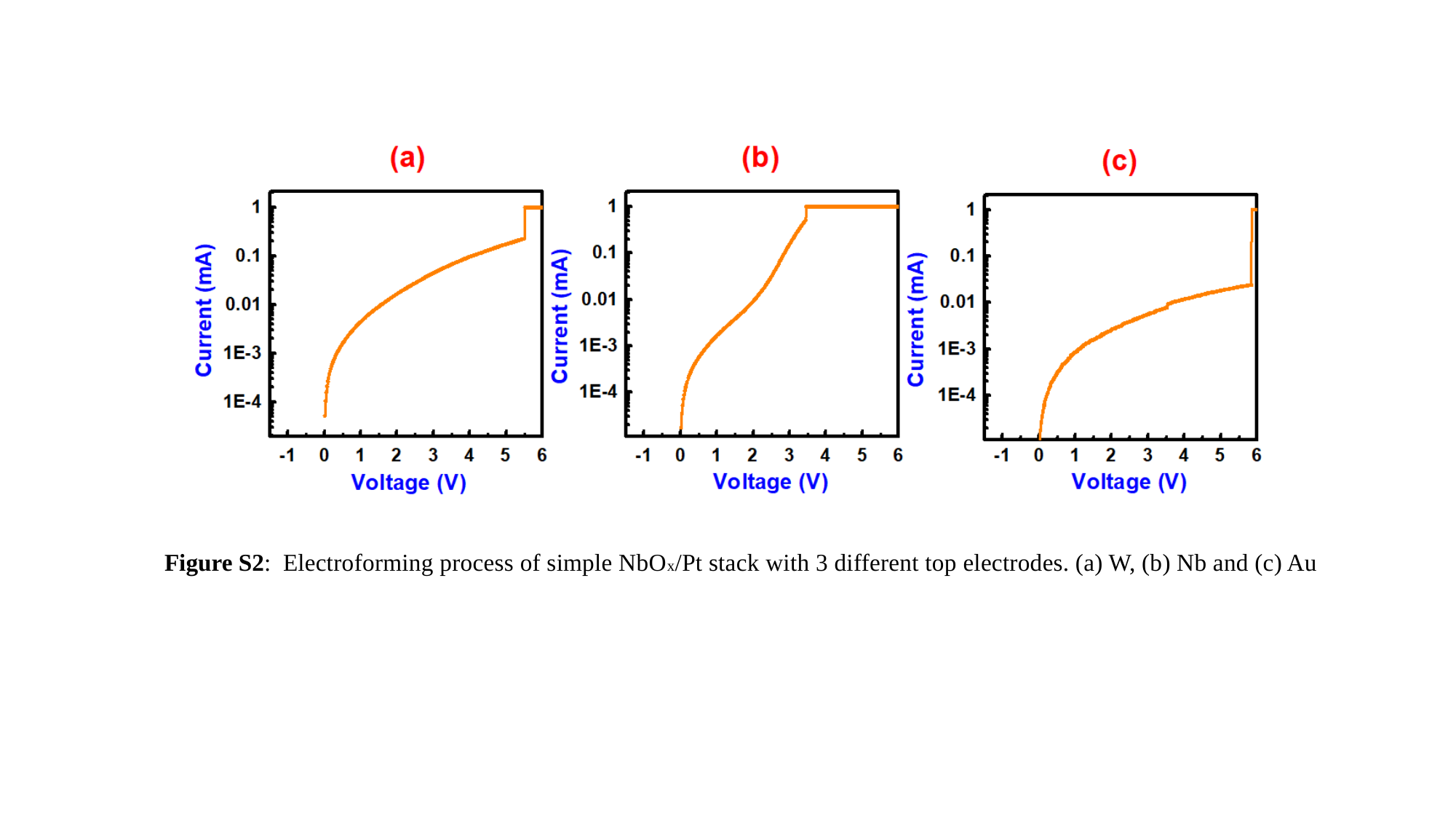

Figure S2: Electroforming process of simple NbOx/Pt stack with 3 different top electrodes. (a) W, (b) Nb and (c) Au

## Slide 4
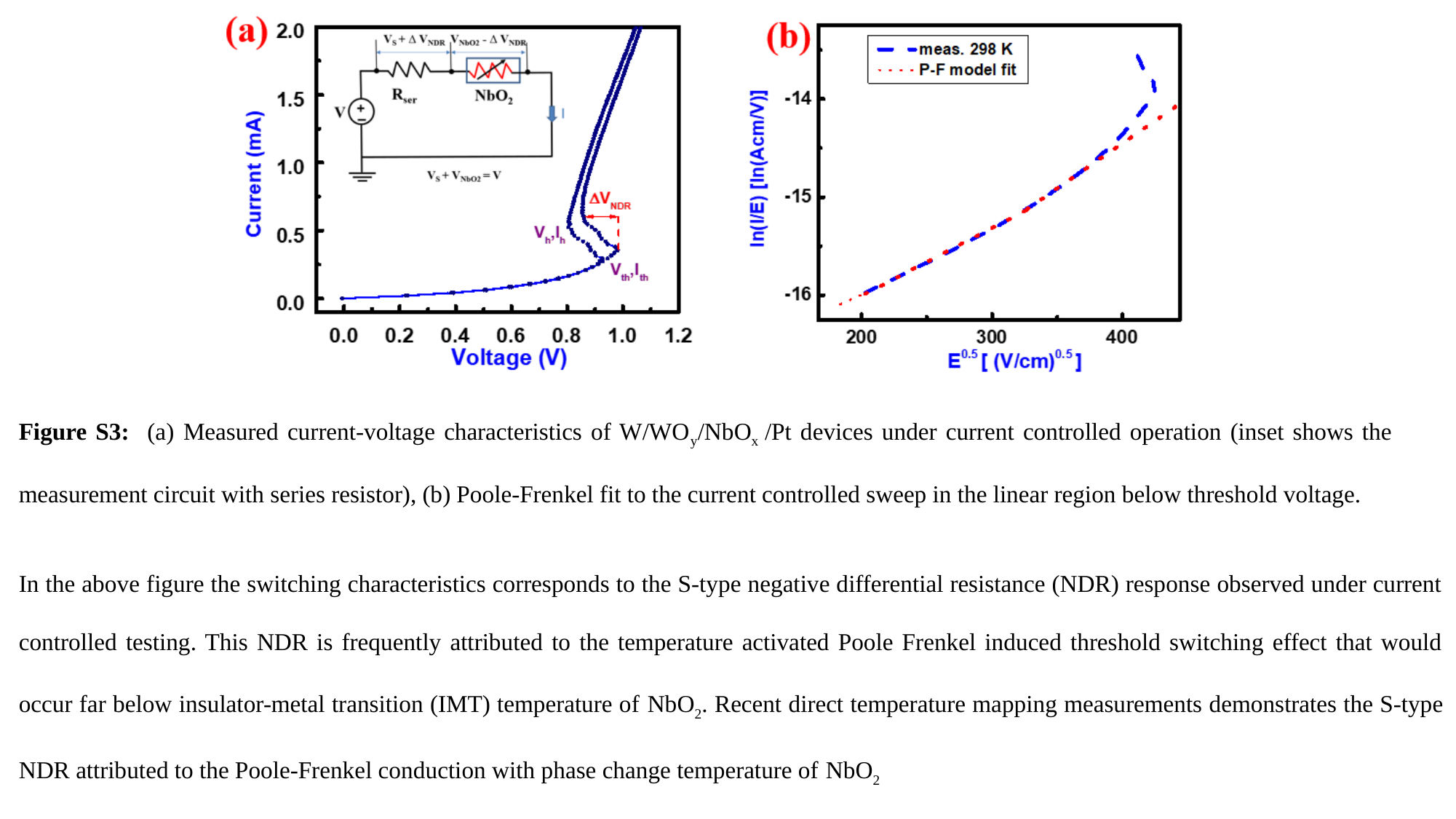

Figure S3: (a) Measured current-voltage characteristics of W/WOy/NbOx /Pt devices under current controlled operation (inset shows the measurement circuit with series resistor), (b) Poole-Frenkel fit to the current controlled sweep in the linear region below threshold voltage.
In the above figure the switching characteristics corresponds to the S-type negative differential resistance (NDR) response observed under current controlled testing. This NDR is frequently attributed to the temperature activated Poole Frenkel induced threshold switching effect that would occur far below insulator-metal transition (IMT) temperature of NbO2. Recent direct temperature mapping measurements demonstrates the S-type NDR attributed to the Poole-Frenkel conduction with phase change temperature of NbO2

## Slide 5
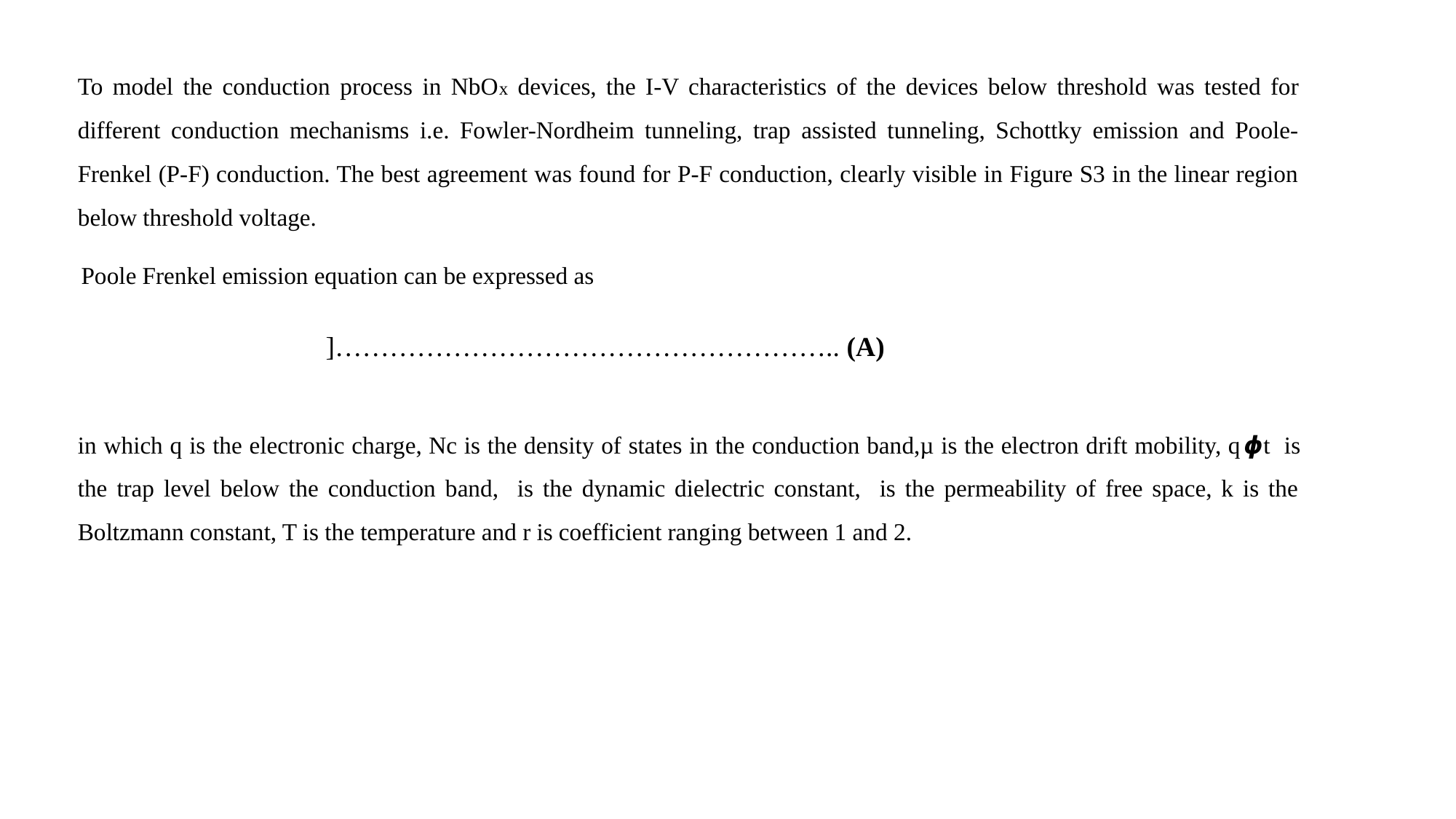

To model the conduction process in NbOx devices, the I-V characteristics of the devices below threshold was tested for different conduction mechanisms i.e. Fowler-Nordheim tunneling, trap assisted tunneling, Schottky emission and Poole-Frenkel (P-F) conduction. The best agreement was found for P-F conduction, clearly visible in Figure S3 in the linear region below threshold voltage.
Poole Frenkel emission equation can be expressed as

## Slide 6
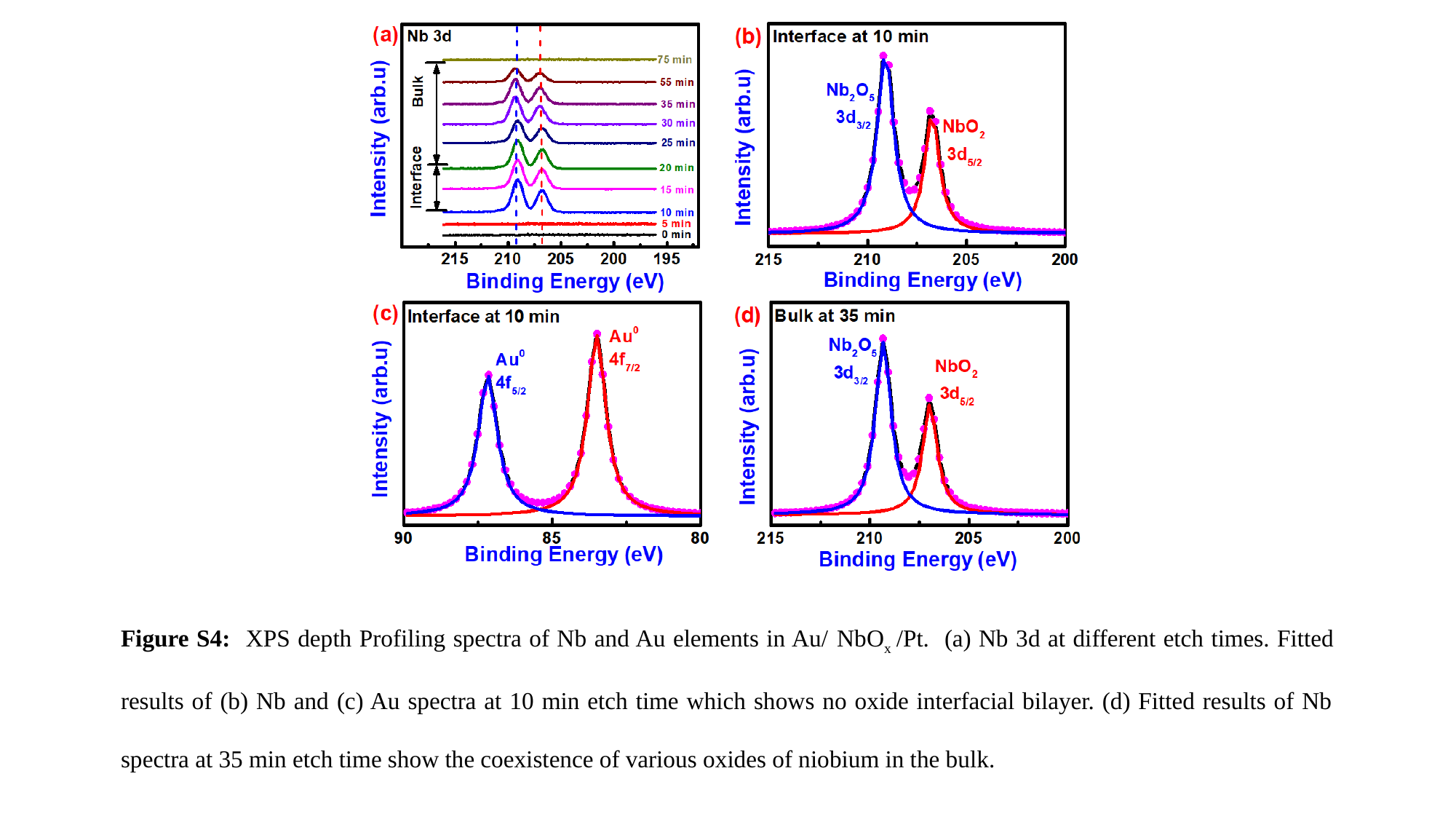

Figure S4: XPS depth Profiling spectra of Nb and Au elements in Au/ NbOx /Pt. (a) Nb 3d at different etch times. Fitted results of (b) Nb and (c) Au spectra at 10 min etch time which shows no oxide interfacial bilayer. (d) Fitted results of Nb spectra at 35 min etch time show the coexistence of various oxides of niobium in the bulk.

## Slide 7
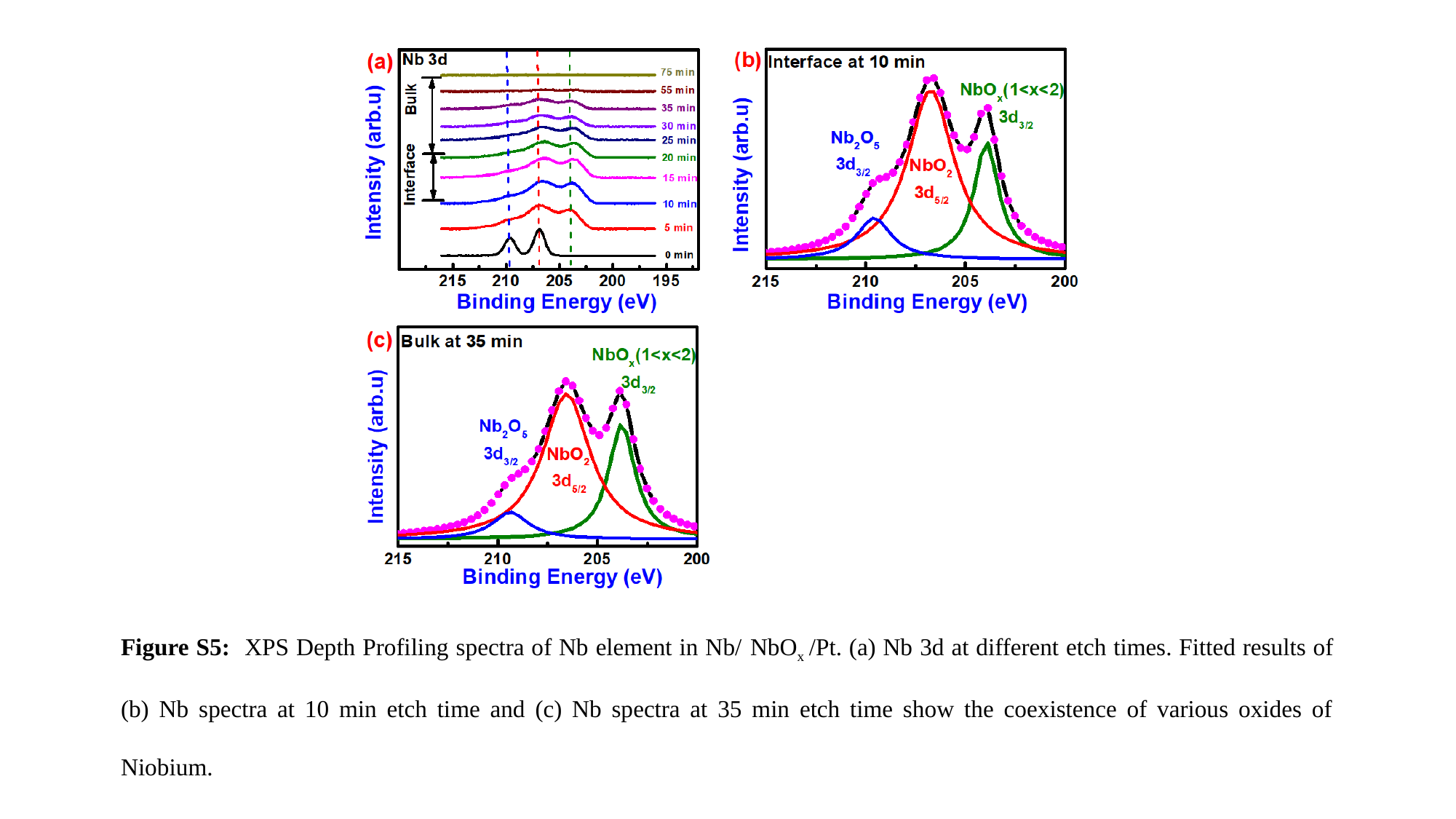

Figure S5: XPS Depth Profiling spectra of Nb element in Nb/ NbOx /Pt. (a) Nb 3d at different etch times. Fitted results of (b) Nb spectra at 10 min etch time and (c) Nb spectra at 35 min etch time show the coexistence of various oxides of Niobium.
